# Effects of Missing Data on Heart Rate Variability Measured From A Smartwatch: Exploratory Observational Study

**DOI:** 10.2196/53645

**Published:** 2025-02-24

**Authors:** Hope Davis-Wilson, Meghan Hegarty-Craver, Pooja Gaur, Matthew Boyce, Jonathan R Holt, Edward Preble, Randall Eckhoff, Lei Li, Howard Walls, David Dausch, Dorota Temple

**Affiliations:** 1 RTI International Morrisville, NC United States

**Keywords:** plethysmography, electrocardiogram, missing data, smartwatch, wearable, ECG, photoplethysmography, PPG, mobile phone, heart rate, pilot study, photoplethysmography, detection, sensor, monitoring, health metric, measure, electrocardiogram, real-world settings, rest, physical activity, remote monitoring, medical setting, youth, adolescent, teen, teenager

## Abstract

**Background:**

Measuring heart rate variability (HRV) through wearable photoplethysmography sensors from smartwatches is gaining popularity for monitoring many health conditions. However, missing data caused by insufficient wear compliance or signal quality can degrade the performance of health metrics or algorithm calculations. Research is needed on how to best account for missing data and to assess the accuracy of metrics derived from photoplethysmography sensors.

**Objective:**

This study aimed to evaluate the influence of missing data on HRV metrics collected from smartwatches both at rest and during activity in real-world settings and to evaluate HRV agreement and consistency between wearable photoplethysmography and gold-standard wearable electrocardiogram (ECG) sensors in real-world settings.

**Methods:**

Healthy participants were outfitted with a smartwatch with a photoplethysmography sensor that collected high-resolution interbeat interval (IBI) data to wear continuously (day and night) for up to 6 months. New datasets were created with various amounts of missing data and then compared with the original (reference) datasets. 5-minute windows of each HRV metric (median IBI, SD of IBI values [STDRR], root-mean-square of the difference in successive IBI values [RMSDRR], low-frequency [LF] power, high-frequency [HF] power, and the ratio of LF to HF power [LF/HF]) were compared between the reference and the missing datasets (10%, 20%, 35%, and 60% missing data). HRV metrics calculated from the photoplethysmography sensor were compared with HRV metrics calculated from a chest-worn ECG sensor.

**Results:**

At rest, median IBI remained stable until at least 60% of data degradation (*P*=.24), STDRR remained stable until at least 35% of data degradation (*P*=.02), and RMSDRR remained stable until at least 35% data degradation (*P*=.001). During the activity, STDRR remained stable until 20% data degradation (*P*=.02) while median IBI (*P*=.01) and RMSDRR *P*<.001) were unstable at 10% data degradation. LF (rest: *P*<.001; activity: *P*<.001), HF (rest: *P*<.001, activity: *P*<.001), and LF/HF (rest: *P*<.001, activity: *P*<.001) were unstable at 10% data degradation during rest and activity. Median IBI values calculated from photoplethysmography sensors had a moderate agreement (intraclass correlation coefficient [ICC]=0.585) and consistency (ICC=0.589) and LF had moderate consistency (ICC=0.545) with ECG sensors. Other HRV metrics demonstrated poor agreement (ICC=0.071-0.472).

**Conclusions:**

This study describes a methodology for the extraction of HRV metrics from photoplethysmography sensor data that resulted in stable and valid metrics while using the least amount of available data. While smartwatches containing photoplethysmography sensors are valuable for remote monitoring of patients, future work is needed to identify best practices for using these sensors to evaluate HRV in medical settings.

## Introduction

Remote patient monitoring using digitally transmitted health data through wearable physiological and activity sensors offers many benefits and is gaining acceptance in the medical community [1]. For example, applications of remote monitoring include acute conditions such as COVID-19 recovery [2] along with chronic conditions such as heart failure [3], chronic obstructive pulmonary disease [4], and diabetes [5]. Remote patient monitoring via wearable sensors can improve clinical outcomes, improve self-management of diseases, and increase patient engagement and satisfaction [6]. Many commercial off-the-shelf (COTS) wearable sensors provide data on important physiological information, particularly wrist-worn devices used to measure heart rate [7,8]. Wrist-worn devices most often use embedded photoplethysmography sensors that detect changes in light intensity on the skin surface due to changes in blood volume during the cardiac cycle to estimate heart rate [9]. While not all smartwatches are certified as medical devices currently, they offer a way of assessing the viability of photoplethysmography sensors, which are used in some cleared medical devices [9-11].

Heart rate variability (HRV) is an indicator of autonomic nervous system functioning and can be used to identify health conditions such as respiratory illness [12], multiple sclerosis [13], Parkinson disease [14], and traumatic brain injury [15]. While collecting wearable sensor data such as HRV can provide multiple benefits to patients and create a more holistic picture of health status, real-world collection of HRV can be difficult to assess due to missing data, differences in data processing techniques, and the quality of data collected [16]. Missing data due to compliance and low-quality signals is an issue when using wearable sensors to monitor HRV in patients in clinical or research settings [16]. Missing data is common in longitudinal research studies, and it is problematic because it can reduce statistical power and cause bias in the estimation of parameters [17]. This is of concern when attempting to identify health anomalies because identifying a change in health relies on consistent and accurate baseline HRV data [18]. As demand for remote patient monitoring grows, an important next step is to identify best practices for data acquisition, processing, and analysis of HRV data from wearable sensors.

In addition to the need for best practices regarding data missingness, more understanding is still needed for accuracy in wrist-worn photoplethysmography sensors compared with more accurate wearable devices. Wrist-worn sensors typically result in greater wear time compared with chest-worn devices; however, wrist-worn sensors are more prone to movement-related artifacts that can negatively influence HRV signal quality [6]. Overwhelmingly, real-world studies have opted to use wrist-worn photoplethysmography sensors to evaluate HRV over other more accurate methods such as chest-worn electrocardiogram (ECG) sensors, likely improving patient compliance in these studies. As a result, we must determine how to best assess data from wrist-worn photoplethysmography sensors, including accounting for missing data and assessing data validity compared with ECG HRV data. This study conducted long-term (months) continuous monitoring of high-resolution HRV data during daily life activities using a COTS smartwatch with a photoplethysmography sensor and activity (step count) data. The primary aim of this study was to evaluate the influence of missing data on HRV metrics collected from a photoplethysmography-based smartwatch both at rest and during physical activity in real-world settings. The secondary aim of this study was to evaluate HRV agreement and consistency between the wrist-worn photoplethysmography sensor and a chest-worn ECG heart rate monitor both at rest and during periods of physical activity and sleep.

## Methods

### Ethical Considerations

For this prospective observational study, wearable sensor data was collected from local community members. The protocol was approved by the Research Triangle Institute (RTI) international institutional review board (MOD00001413). Written consent was received from all participants for participation and data use. The consent process emphasized that participation was voluntary and that individuals were allowed to withdraw their consent for participation at any time. Personal identifying information was stored in a secured file and kept separate from data collected once enrolled in the study. All smartwatch and survey data were deidentified and labeled by a participant identifier for analysis. Participants were offered US $50 every 3 months as study compensation, and participants could earn up to US $100 if they were enrolled for the entire 6 months of the study duration.

### Participants

Healthy volunteers aged 18 years and older were included in this study. Individuals who were pregnant or who had a pre-existing health condition were not excluded from participation if they were able to use the wearable sensor. Participants were enrolled for 3 months with the opportunity to re-enroll for up to 6 months. Demographic data were obtained and stored in Research Electronic Data Capture (REDCap). Recruitment and data collection took place between May 2022 and June 2023.

### Device Protocol

Each participant was outfitted with a Garmin Fenix 6/6s smartwatch, which was worn on the nondominant wrist. We chose to use Garmin over other wrist-worn photoplethysmography sensors because its Health Companion Software Development Kit provides access to high-resolution interbeat interval (IBI) data collected by photoplethysmography sensors that allow for monitoring of HRV. Other COTS sensors only provide summary and other processed data. The average heart rate derived from Garmin watches strongly correlates with clinical ECG heart rate data (*r*=0.96) [19]; however, the accuracy of HRV metrics derived from Garmin has yet to be studied. Garmin watches integrate 2 photoplethysmography sensors for monitoring HR and a 3-axis accelerometer for detecting movement in the x, y, and z directions. The watch firmware uses accelerometer signals to calculate step counts in 1-minute windows. We used the Companion Software Development Kit to access minimally processed IBI data and step counts from the participants. The SIGMA+ Health app (S+ app) was installed onto the participant’s personal mobile device or a study-issued Samsung Galaxy 12 smartphone if participants preferred not to use their personal mobile device for the study. The Garmin smartwatch was paired through a Bluetooth low energy connection to the mobile device and data from the watch was ingested through the S+ app onto an RTI secure Amazon Web Services server in JavaScript Object Notation format for offline data cleaning, metric extraction and standardization, and analysis. The initial setup included giving each user the study Personal identification number and a unique participant ID that did not contain personally identifiable information, which allowed the S+ app to track the user’s data from one or more devices through the duration of the study. Within the app, the participant could view the connectivity status of their Garmin Fenix watch and manually trigger a data sync if required. Participants completed a daily health survey administered within the S+ app, which asked questions related to illness symptoms, sleep quality, sleep duration, and any unusual events that may have occurred that day (ie, psychological stress, excessive exercise, etc). Results were stored in a REDCap database.

A subset of participants (n=5) wore a Polar H10 ECG heart rate monitor with a sampling frequency of 1000 Hz over their chest simultaneously with the Garmin smartwatch to assess agreement and consistency between HRV metrics. IBI values derived from the Polar H10 chest strap show strong agreement compared with clinical ECG recordings at rest (intraclass correlation coefficient [ICC]=0.95) and with light exercise (ICC=0.93) [20]. Participants were encouraged to wear the Polar H10 continuously (day and night) for 5 days and were instructed to only remove it during showers, baths, or swimming. Polar H10 data was retrieved through the Elite HRV mobile app, which was downloaded to the participant’s personal smartphone before data collection [21]. Excel files containing raw IBI data were exported from the participant’s phone and sent to an RTI email account. We did not get measures of step counts from the Polar H10 device because Elite HRV only collects HRV data.

### Data Processing for IBI Data From Garmin and Polar Devices

Each IBI data point acquired through the Garmin smartwatch and S+ app had an associated timestamp with millisecond precision. Steps from the Garmin were reported at 60-second intervals and IBI values were reported with each detected beat. Each IBI data point acquired through the Polar H10 monitor and Elite HRV app was recorded with millisecond precision. IBI artifacts (IBI>1500 ms or IBI <300 ms) were flagged and removed. For Garmin data quality control, we evaluated the expected data fraction, calculated as a ratio of the number of incoming data points to the maximum number of data points expected during the monitoring period, to assess data missingness. For simplicity, we assumed the expected number of IBI data points would be 60 IBI values/minute. This would correlate with an average heart rate of 60 beats per minute, which is a typical heart rate for someone who is sitting or resting. The exact number of data points expected varied due to within- and between-person differences in average heart rate. The expected data fraction was multiplied by 100 to be represented as a percentage between 0 and 100. For analysis, we chose to evaluate Garmin datasets with at least 70% of expected data to ensure we were using high-quality datasets with minimal missing data points. The first approximately 2 weeks of data were selected for each participant with at least 70% expected data fraction. Although there is some variation in the number of IBI data points in each 5 min window (a higher heart rate results in more IBI data points within a window), the datasets with at least 70% of expected data that were used for analysis contained a mean of 250 (SD 126) IBI data points within each 5 min window.

Garmin and Polar data were processed into 5-minute windows with each point in the window formatted as (timestamp, value) and aligned based on real-time so that the windows start at 5-minute marks (eg, 12:00 AM, 12:05 AM). The following metrics were extracted in 5 min windows: time-domain metrics (median IBI, SD of IBI values [STDRR], root-mean-square of the difference in successive IBI values [RMSDRR[) and frequency-domain metrics (low-frequency [LF] power, high-frequency [HF] power, and ratio of LF to HF power [LF/HF]) [18,22]. To calculate HRV metrics in the frequency domain, we resampled IBI data to 5 Hz using the Piecewise Cubic Hermite Interpolating Polynomial method [23]. If there was a gap in the signal greater than 15s, the longest continuous segment in the 5-minute window was used. If there were more than 3 gaps of this length, we did not use this window. After interpolation, the signal was filtered to the low-frequency (LF: 004 to 0.15 Hz) and high-frequency regions (HF: 0.15 to 1.0 Hz; we increased the upper limit to 1.0 Hz vs the typically used 0.40 Hz to account for physical activity when breathing rates and heart rates increase [24,25]). We computed a value representing the LF and HF power for 60-second and 30-second windows, respectively, using the formula: value = ln (variance [data window]). Values less than 2.5 and more than 9.0 were eliminated, and the median values were calculated to represent the 5-minute window. For Garmin smartwatch step data, reported every minute, we calculated the average of values in the 5-minute window; we required at least 2 valid data points for the calculation.

### Semisimulation of Garmin Data Degradation

A semisimulation design based on previous work [26] was used to create new datasets with various amounts of Garmin IBI data from original Garmin datasets. The premise of this semisimulation approach was to create new datasets with different amounts of missing data based on real wear patterns. Using this method, we ensured that we removed data in a realistic manner rather than randomly removing data. In this approach, we considered data missingness patterns from datasets with low expected data fractions and applied these patterns to the datasets with high expected data fractions. These semisimulated datasets were then compared to the reference datasets to understand how varying amounts of data missingness influence HRV metrics. In total, 4 additional 5-minute window samples were randomly selected that contained fewer IBI data points. These 4 samples were inspected to identify data gap patterns and then used to model data missingness patterns in the selected datasets.

To recreate data missingness, we matched the data missingness pattern from the sample 5-minute windows to each 5-minute window in the reference datasets used for analysis. For example, one sample dataset with 120 IBI data points (missing 60% of data) was missing data in the first 3.15 min of the window. The first 3.15 min of each 5-min window was removed from the reference dataset to simulate data missingness. The simulated datasets were then compared with the reference datasets to identify differences in HRV metrics. Missing data simulations were performed using Python (Python Software Foundation) [27]. The various datasets were labeled for analysis, that consists of (1) a reference dataset with no missing data, (2) missing 10% of data, (3) missing 20% of data, (4) missing 35% of data, and (5) missing 60% of data. No simulated data degradation was performed for Polar H10 IBI data.

### Statistical Analysis

**Figure 1:**
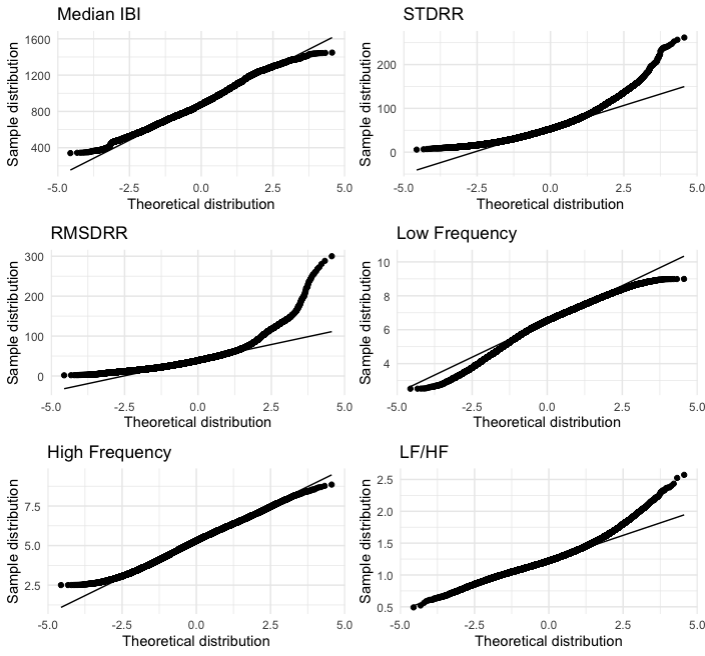
Quantile-Quantile plots for the median interbeat interval (IBI), SD of IBI values (STDRR), root-mean-square error of IBI values (RMSDRR), low frequency (LF), high frequency (HF), and the ratio of low to high frequency power at rest (steps/min=0). Box-Cox transformations were performed for STDRR and RMSDRR metrics. Smartwatch IBI data was collected and reported from 16 individuals recruited from a community setting between May 2022 and June 2023.

**Figure 2:**
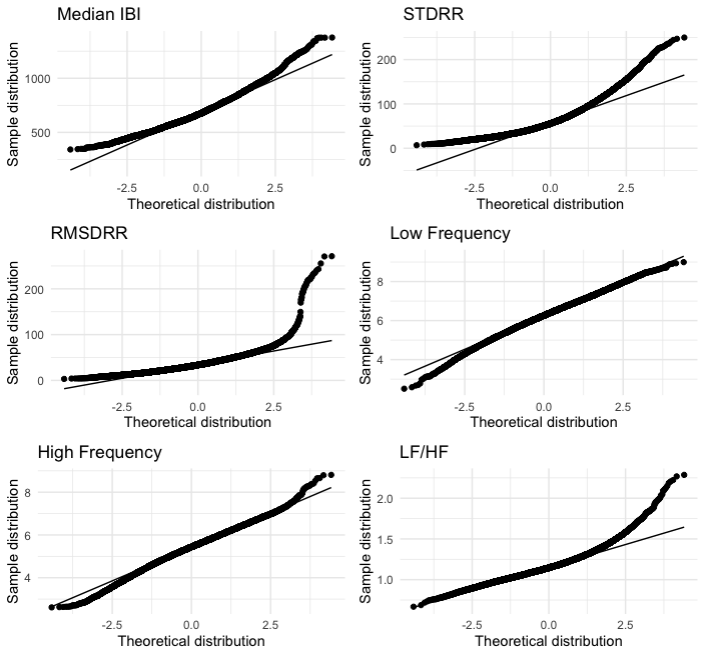
Quantile-Quantile plots for the median interbeat interval (IBI), SD of IBI values (STDRR), root-mean-square error of IBI values (RMSDRR), low frequency (LF), high frequency (HF), and the ratio of low to high frequency power at light activity (100>steps/min>0). Box-Cox transformations were performed for RMSDRR and LF/HF metrics. Smartwatch IBI data were collected and reported from 16 individuals recruited from a community setting between May 2022 and June 2023.

Mean and SD were calculated for all descriptive variables and HRV metrics included in the analysis. For the primary aim, due to the large sample size of Garmin 5-minute windows for rest (48,442 windows) and light activity (19,976 windows), Shapiro-Wilk normality tests were not valid; therefore, we visually inspected each Garmin HRV metric for normality using Quantile-Quantile (Q-Q) plots. Q-Q plots that appeared as a roughly straight 45-degree line were considered normally distributed. Variables that were not normally distributed were transformed using a Box-Cox transformation before analysis. Q-Q plots of each HRV variable are shown at rest in Figure 1 and at light activity in Figure 2.

We then used linear mixed-effect models to estimate the mean differences in each Garmin HRV metric between the datasets with missing samples by level of missingness and the reference dataset. A participant-specific random intercept was included in the models to account for correlations of our sample data among multiple 5-minute windows within each participant [28]. An indicator of the level of data missingness (data without missing samples as reference, data with 10%, 20%, 35%, or 60% samples removed) was included in the models as a fixed-effect covariate. The Ime4 package in the statistical environment R (R Core Team) was used for the model estimations [29]. The statistical significance of the estimated differences was tested using Satterthwaite’s method with a 2-tailed significance level of 0.05, and the 95% CI were obtained using the bootstrap method through the bootMer function of the Ime4 package in R (1000 simulations). Separate linear mixed models were estimated using sample data under 2 different conditions: at rest (steps/min=0) and during light physical activity (steps/min of >0 and <100).

For the secondary aim, we assessed the reliability of Garmin-derived HRV metrics within each participant and their validity compared to Polar-derived HRV metrics. The intraclass correlation coefficients (class 2, mean rating; ICC_2,k_) for consistency and absolute agreement with 95% CI were obtained using the irr package in the statistical environment R [30]. The magnitude of the ICC was generally interpreted as ICC_2,k_ of 0.5-0.75 as “moderate”, ICC_2,k_ >0.75 as “good”, and ICC_2,k_ >0.9 as “excellent” [31]. In addition, we visually assessed agreement between Garmin-derived HRV metrics and polar-derived HRV metrics using Bland-Altman analyses to evaluate the mean differences and 95% limits of agreement [32]. R version 4.2.2 [33] was used for statistical analyses.

## Results

### Overview

Garmin data were collected from 31 participants for up to 180 days. The analyzed sample in aim 1 used a mean of 16 (SD 3) consecutive days of data from 16 participants resulting in 48,442 five-minute windows of data that represented rest (steps/min=0) and 19,976 windows of data that represented light activity (100 >steps/min >0). Although there is not a clear precedence for what percentage of missing data is acceptable, we wanted the reference datasets to contain a minimal amount of missing data. Therefore, each participant included in the analysis had an expected data fraction of at least 70% (including daytime and nighttime), and participants who did not meet this requirement (n=15) were not included in the analysis. Differences in days of data occurred because of between-person variance in unprocessed IBI data and differences in wear patterns. For aim 2, 5 participants wore both the Garmin Fenix watch and Polar heart rate monitor for 5 consecutive days. Demographic data is shown in Table 1. The averages of each HRV metric for aims 1 and 2 are shown in Table 2 and Table 3. Box-Cox transformations were performed for STDRR and RMSDRR metrics at rest (steps/min=0; Figure 1) and RMSDRR and LF/HF at light activity (100>steps/min>0; Figure 2). Tables 4 and 5 show results from the linear mixed models, specifically the estimated error between the reference and missing data, the SE, and the 95% CIs of the estimated difference.

**Table 1:** Participant characteristics of a community sample of individuals who provided smartwatch data between May 2022 and June 2023. In total, 31 individuals were recruited, data from 16 individuals were used in aim 1, and data from 5 individuals were used in aim 2.

	Total sample (n=31), mean (SD)	Aim 1 (n=16), mean (SD)	Aim 2 (n=5), mean (SD)
Age (years)	38.6 (10.7)	38.6 (13.3)	35.2 (4.1)
Sex (females), n (%)	16 (53)	13 (81)	3 (60)
Height (m)	1.73 (0.12)	1.77 (0.12)	1.77 (0.08)
Mass (kg)	83.4 (17.2)	87.1 (16.5)	95.4 (24.7)
BMI (kg/m^2^)	27.9 (4.7)	27.7 (4.1)	30.2 (5.6)

**Table 2:** Heart rate variability metrics for aim 1 datasets in a community sample of 16 individuals who provided smartwatch data between May 2022 and June 2023.

		Reference Garmin data	10% Missing Garmin data	20% Missing Garmin data	35% Missing Garmin data	60% Missing Garmin data
**Resting (steps/min=0)**						
	Median interbeat interval	888.86	888.17	887.88	888.45	887.85
	SD of interbeat interval values	57.41	57.44	57.44	57.14	52.87
	Root-mean-square of the difference in successive interbeat interval values	41.70	41.83	41.86	41.53	40.98
	Low-frequency power	6.52	6.38	6.31	6.50	6.50
	High-frequency power	5.51	5.01	4.83	5.50	5.51
	Ratio of low-frequency to high-frequency power	1.19	1.29	1.32	1.20	1.20
**Light activity (0<steps/min<100)**						
	Median interbeat interval	690.70	688.02	687.19	692.65	695.30
	SD of interbeat interval values	63.90	63.55	63.39	63.96	50.31
	Root-mean-square of the difference in successive interbeat interval values	35.20	35.72	35.87	35.11	34.57
	Low-frequency power	6.28	6.22	6.19	6.26	6.26
	High-frequency power	5.59	5.26	5.13	5.55	5.55
	Ratio of low-frequency to high-frequency power	1.13	1.19	1.21	1.13	1.14

**Table 3:** Heart rate variability metrics for aim 2 datasets in a community sample of 5 individuals who provided smartwatch data between May 2022 and June 2023.

		Reference Garmin data	10% Missing Garmin data	20% Missing Garmin data	35% Missing Garmin data	60% Missing Garmin data
**Resting (steps/min=0)**						
	Median interbeat interval	888.86	888.17	887.88	888.45	887.85
	SD of interbeat interval values	57.41	57.44	57.44	57.14	52.87
	Root-mean-square error	41.70	41.83	41.86	41.53	40.98
	Low-frequency power	6.52	6.38	6.31	6.50	6.50
	High-frequency power	5.51	5.01	4.83	5.50	5.51
	Ratio of low-frequency to high-frequency power	1.19	1.29	1.32	1.20	1.20
**Light activity (0<steps/min<100)**						
	Median interbeat interval	690.70	688.02	687.19	692.65	695.30
	SD of interbeat interval values	63.90	63.55	63.39	63.96	50.31
	Root-mean-square error	35.20	35.72	35.87	35.11	34.57
	Low-frequency power	6.28	6.22	6.19	6.26	6.26
	High-frequency power	5.59	5.26	5.13	5.55	5.55
	Ratio of low-frequency to high-frequency power	1.13	1.19	1.21	1.13	1.14

**Table 4:** Linear mixed-effects model results of the effects of missing raw data on heart rate variability metrics collected at rest (steps/min=0) in a community sample of 16 individuals who provided smartwatch data between May 2022 and June 2023.

Heart rate variability metric and level of missing data compared with reference		Estimated difference between reference data and missing data	SE	Lower limit of the 95% CI	Upper limit of the 95% CI	*P* value
**Median** **interbeat interval**						
	10%	–0.739	0.939	–2.643	1.139	.43
	20%	–1.04	0.939	–3.019	0.978	.27
	35%	–0.468	0.939	–2.341	1.374	.62
	60%	–1.095	0.939	–2.880	0.928	.24
**SD of interbeat interval values**						
	10%	–0.002	0.006	–0.014	0.010	.69
	20%	–0.003	0.006	–0.015	0.008	.59
	35%	–0.020	0.006	–0.031	–0.008	.001^a^
	60%	–0.215	0.006	–0.228	–0.204	<.001^a^
**Root-mean-square**						
	10%	0.005	0.004	–0.003	0.013	.27
	20%	0.005	0.004	–0.003	0.014	.20
	35%	-0.014	0.004	–0.021	–0.005	.001^a^
	60%	-0.053	0.004	–0.061	–0.044	<.001^a^
**Low-frequency power**						
	10%	-0.134	0.006	–0.145	–0.122	<.001^a^
	20%	-0.204	0.006	–0.215	–0.193	<.001^a^
	35%	-0.016	0.006	–0.028	–0.005	.005^a^
	60%	-0.016	0.006	–0.028	–0.005	.004^a^
**High-frequency power**						
	10%	-0.501	0.005	–0.512	–0.490	<.001^a^
	20%	-0.678	0.005	–0.689	–0.667	<.001^a^
	35%	-0.013	0.005	–0.024	–0.002	.02^a^
	60%	-0.006	0.005	–0.016	0.005	.27
**Ratio of low frequency to high frequency**						
	10%	0.096	0.001	0.094	0.098	<.001^a^
	20%	0.130	0.001	0.128	0.132	<.001^a^
	35%	0.005	0.001	0.003	0.007	<.001^a^
	60%	0.004	0.001	0.002	0.006	<.001^a^

^a^*P* value <.05 indicates a significant difference compared with the reference dataset.

**Table 5:** Linear mixed-effects model results of the effects of missing raw data on heart rate variability metrics collected at light activity (100>steps/min>0) in a community sample of 16 individuals who provided smartwatch data between May 2022 and June 2023.

Heart rate variability metric and level of missing data compared with reference		Estimated difference between reference data and missing data	SE	Lower limit of the 95% CI	Upper limit of the 95% CI	*P* value
**Median** **interbeat interval**						
	10%	–2.645	1.049	–4.638	–0.627	.01^a^
	20%	–3.473	1.049	–5.454	–1.481	<.001^a^
	35%	1.998	1.049	–0.051	3.958	.06
	60%	4.631	1.050	2.513	6.577	<.001^a^
**SD of interbeat interval values**						
	10%	–0.005	0.003	–0.011	0.002	.11
	20%	–0.008	0.003	–0.014	–0.001	.02^a^
	35%	–0.006	0.003	–0.012	0.001	.08
	60%	–0.217	0.003	–0.223	–0.210	<.001^a^
**Root-mean-square**						
	10%	0.039	0.007	0.026	0.052	<.001^a^
	20%	0.050	0.007	0.037	0.064	<.001^a^
	35%	–0.013	0.007	–0.025	–.001	.05
	60%	–0.069	0.007	–0.081	–0.055	<.001^a^
**Low-frequency power**						
	10%	–0.054	0.006	–0.067	–0.042	<.001^a^
	20%	–0.087	0.006	–0.100	–0.075	<.001^a^
	35%	–0.027	0.006	–0.040	–0.015	<.001^a^
	60%	–0.019	0.006	–0.031	–0.006	.003^a^
**High-frequency power**						
	10%	–0.324	0.006	–0.336	–0.313	<.001^a^
	20%	–0.452	0.006	–0.464	–0.441	<.001^a^
	35%	–0.033	0.006	–0.045	–0.021	<.001^a^
	60%	–0.038	0.006	–0.050	–0.027	<.001^a^
**Ratio of low frequency to high frequency**						
	10%	0.043	0.004	0.042	0.045	<.001^a^
	20%	0.059	0.001	0.058	0.061	<.001^a^
	35%	–0.001	0.001	–0.003	0.0004	0.12
	60%	–0.001	0.001	–0.001	0.002	0.33

^a^*P* value <0.05 indicates a significant difference compared to the reference dataset.

### Aim 1: Heart Rate Variability Metrics at Rest

**Figure 3:**
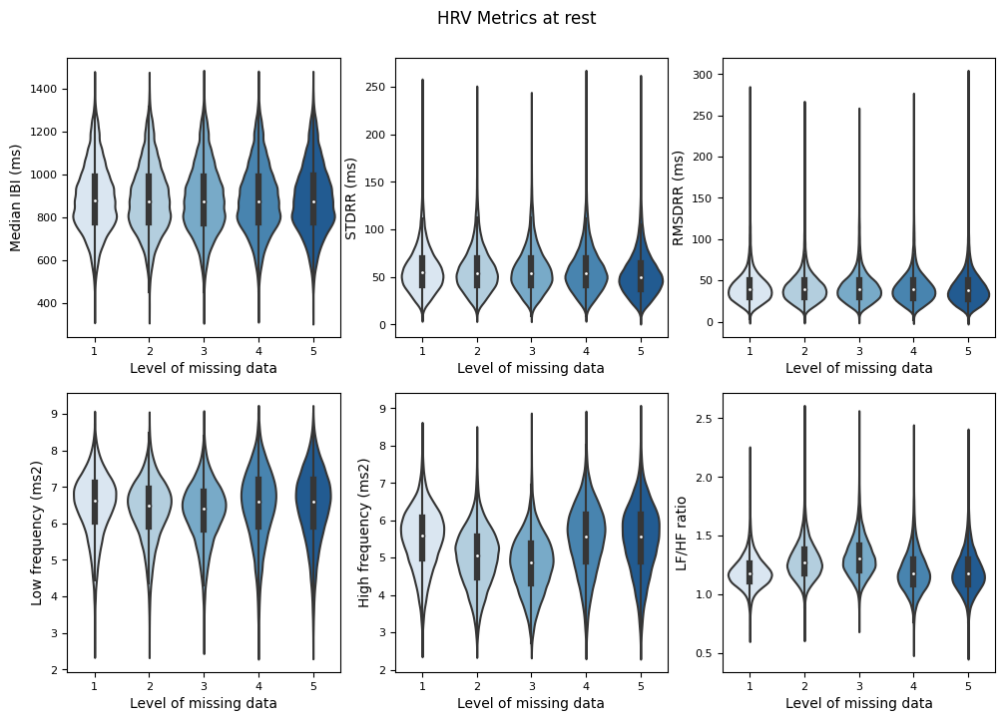
Heart rate variability metrics at rest (steps/min=0). HRV: heart rate variability; IBI: interbeat interval; LF/HF: ratio of low frequency power to high frequency power; RMSDRR: root-mean-square of the difference in successive IBI values; STDRR: standard deviation of interbeat interval values.

At rest (steps/min=0), there was no difference in median IBI values between reference and simulated data (*P*>.05; Table 4 and Figure 3). There were significant differences in STDRR and RMSDRR values between the reference and 35% and 60% missing data (*P*<.05; Table 4, Figure 3). LF and LF/HF metrics demonstrated significant differences between the reference and all simulated datasets (*P*<.05; Table 4, Figure 3). HF in the reference dataset was significantly higher compared to the 10%, 20%, and 35% missing datasets (*P*<.05; Table 4, Figure 3). There was no significant difference between the reference dataset and the 60% missing dataset (*P*>.05; Table 4 and Figure 3).

In Figure 3, reference smartwatch data were compared to (1) 10% missing data (2), 20% missing data (3), 35% missing data (4), and 60% missing data (5) per 5-minute window. Smartwatch IBI data were collected and reported from 16 individuals recruited from a community setting between May 2022 and June 2023.

### Aim 1: Heart Rate Variability Metrics at Light Activity

**Figure 4:**
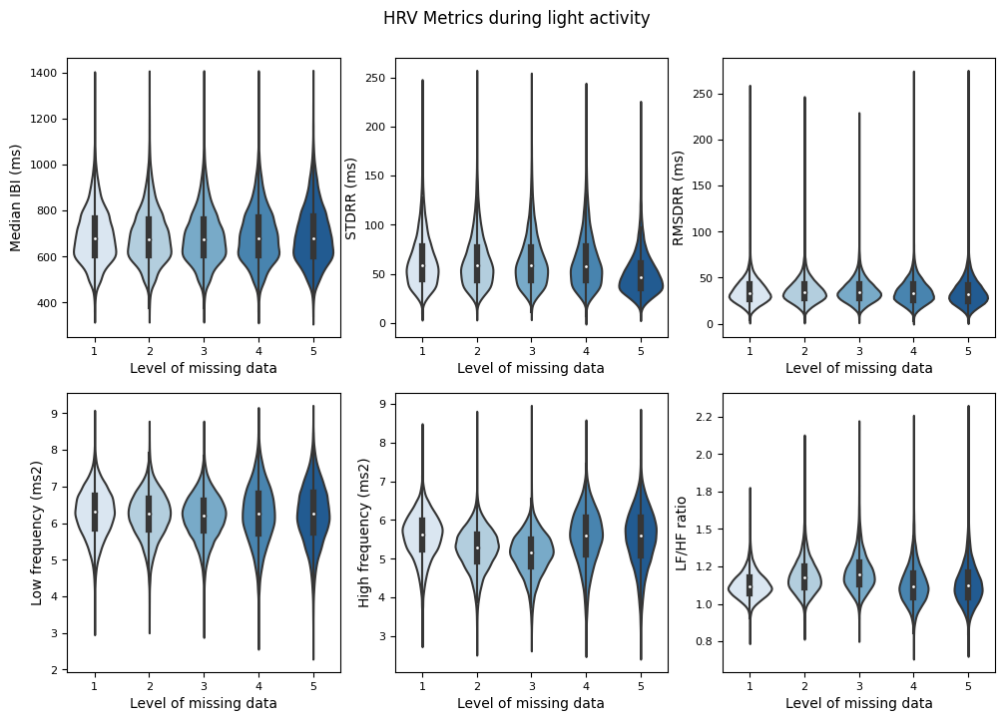
Heart rate variability metrics at light activity (100>steps/min>0). HRV: heart rate variability; IBI: interbeat interval; LF/HF: ratio of low frequency power to high frequency power; RMSDRR: root-mean-square of the difference in successive IBI values; STDRR: standard deviation of interbeat interval values.

At light activity (100>steps/min>0), there were significant differences in median IBI between the reference and all simulated datasets (*P*<.05; Table 5 and Figure 4). STDRR and RMSDRR values were different between the reference and simulated datasets with 10%, 20%, and 60% missing data (*P*<.05; Table 5 and Figure 4). Differences between the reference dataset and 35% missing data were not statistically significant, although results trended toward statistical significance (*P*=.06). LF and HF metrics demonstrated significant differences between the reference dataset and all simulated datasets (*P*<.05; Table 5 and Figure 4). LF/HF in the reference dataset was significantly higher compared with the 10% and 20% missing datasets (*P*<.05; Table 5 and Figure 4). There was no significant difference between the reference dataset and the 35% and 60% missing datasets (*P*>.05; Table 5 and Figure 4).

In Figure 4, Reference smartwatch data were compared to (1) 10% missing data (2), 20% missing data (3), 35% missing data (4), and 60% missing data (5) per 5 min window. Smartwatch IBI data was collected and reported from 16 individuals recruited from a community setting between May 2022 and June 2023.

### Aim 2: Validity and Agreement of Photoplethysmography-Derived Heart Rate Variability Compared With ECG-Derived Heart Rate Variability

**Figure 5:**
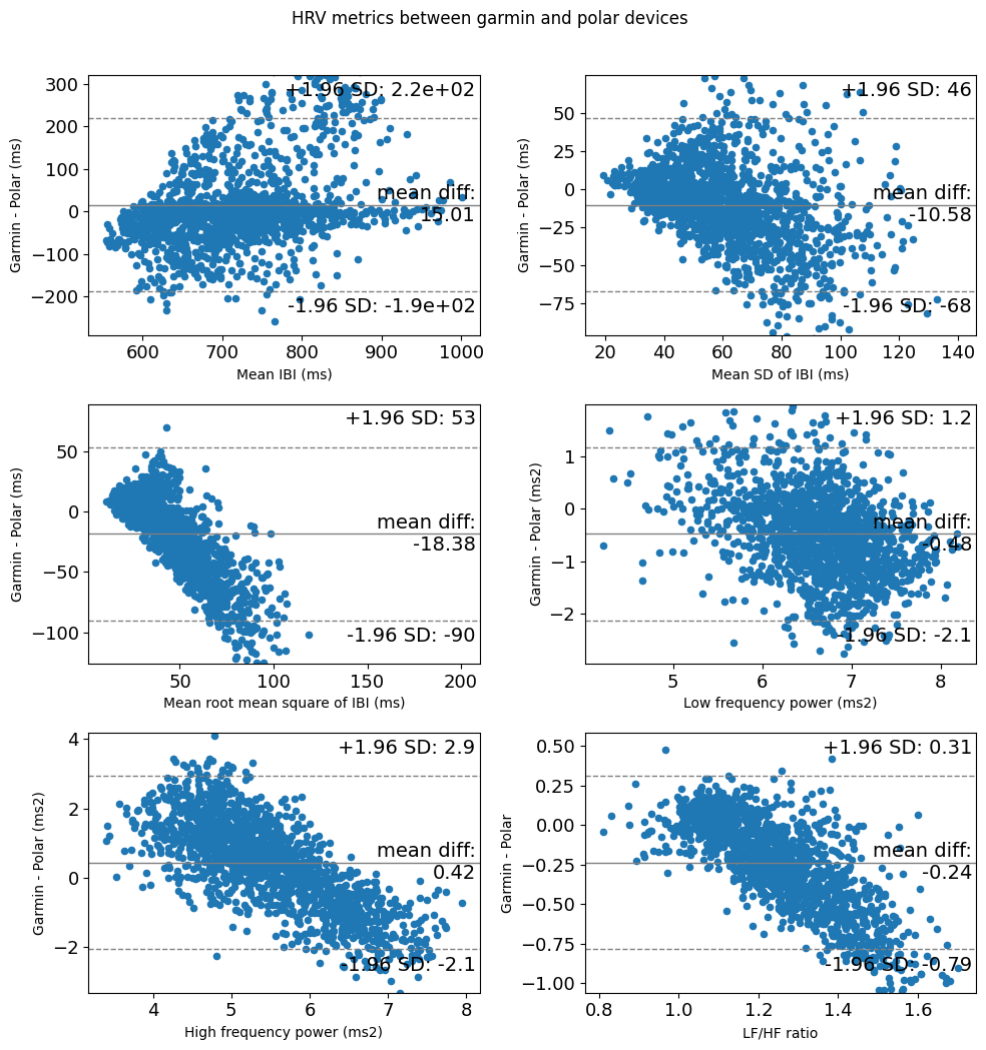
Bland-Altman plots for differences in heart rate variability metrics measured with a Garmin smartwatch and Polar heart rate monitor. HRV data was collected and reported from 5 individuals recruited from a community setting between May 2022 and June 2023. HRV: heart rate variability; IBI: interbeat interval; LF/HF: ratio of low frequency power to high frequency power.

Median IBI values calculated from Garmin photoplethysmography sensors had moderate agreement (ICC=0.585) and consistency (ICC=0.589) with Polar ECG data, and LF calculated from Garmin photoplethysmography sensors had moderate consistency (ICC=0.545) with Polar ECG data. All other HRV metrics (STDRR, RMSDRR, LF, HF, and LF/HF) demonstrated poor agreement between Garmin photoplethysmography and Polar ECG data (ICC=0.071-0.470, Table 6). Bland-Altman visual analysis indicated systematic bias present in all HRV metrics, with particularly notable bias in RMSDRR, HF, and LF/HF (Figure 5). The root-mean-square error between Garmin and Polar devices for each HRV metric are listed in Table 6.

**Table 6:** Agreement and consistency between heart rate variability metrics were measured with a Garmin smartwatch and Polar heart rate monitor in a community sample of 5 individuals who provided data between May 2022 and June 2023.

Heart rate variability metric	ICC^a^ agreement, mean (95% CI)	ICC consistency, mean (95% CI)	Root-mean-square error
Median interbeat interval	0.585 (0.538 to 0.626)	0.589 (0.547 to 0.628)	105.23
SD of interbeat interval values	0.441 (0.302 to 0.545)	0.472 (0.417 to 0.521)	30.92
Root mean square of the difference in successive interbeat interval values	0.214 (0.028 to 0.357)	0.255 (0.178 to 0.324)	40.98
Low-frequency power	0.473 (0.163 to 0.645)	0.545 (0.497 to 0.588)	0.97
High-frequency power	0.404 (0.283 to 0.499)	0.429 (0.370 to 0.483)	1.35
Low-frequency to high-frequency power ratio	0.071 (–0.09 to 0.207)	0.117 (0.024 to 0.2)	0.37

^a^ICC: intraclass correlation coefficient.

## Discussion

### Principal Findings

To our knowledge, this paper shows for the first time the results of long-term (months) continuous monitoring of high-resolution HRV data during daily life activities using a COTS smartwatch with photoplethysmography sensor and activity (step count) data. This study describes a methodology for the extraction of HRV metrics from IBI time-series data that resulted in stable and valid metrics while using the least amount of available data. By understanding patterns of missing data, we can maximize the amount of usable data and minimize the impact of data gaps due to suboptimal wear compliance or any issues in the data synchronization between the watch and the smartphone. We found that time-domain HRV metrics (median IBI, STDRR, and RMSDRR) are more resilient to missing data when the participant is in a resting state; however, during light activity missing data influence time-domain HRV metrics. Median IBI measurements remain stable at rest even at 60% data degradation. STDRR and RMSDRR measurements remain stable at rest until 35% data degradation. Frequency-domain HRV metrics (LF, HF, and LF/HF) are less resilient to missing data both at rest and during light activity and are unstable even at 10% data degradation. It is unclear why HF (during rest) was not significantly different at 60% data degradation and LF/HF (during light activity) was not significantly at 35% and 60% data degradation compared to reference data. We speculate that there may not be enough data at these levels of data degradation for accurate comparison. Results from this study suggest that analyses and algorithms that use primarily frequency-domain HRV metrics are more sensitive to sparse data collection. Researchers and engineers should carefully evaluate HRV metrics in situations where data are sparse. If missing data are an issue in a research study, it may be beneficial to rely on metrics and algorithms using time-domain HRV metrics.

A few previous studies have simulated missing data patterns in HRV data by evaluating differences between scattered missing beats (one or two missing data points dispersed throughout the dataset) and bursts of missing beats (longer periods of missing data likely due to not wearing the device) [34,35]. Our study considered data characteristics from real HRV data patterns to remove data instead of using a random data removal approach. We found that datasets with very sparse data (missing 35% and 60% of data points per 5-minute window) typically had one burst of missing data. For example, we identified a 3.15 min data gap (60%) during the beginning of one 5-minute window. We also identified missing data between 1 minute and 2.75 minutes (35%) of another 5-minute window. The 20% and 10% missing datasets were missing 0.80 minutes and 0.63 minutes, respectively, of data scattered randomly throughout the trial, most often in 1-2 second increments. In general, 5 min windows that had data gaps that were smaller and more randomly dispersed had a greater number of IBI datapoints, whereas 5-minute windows that had one larger burst of missing data, often at the beginning of the 5-minute window, had fewer IBI datapoints. We hypothesize that these larger bursts of missing data may be the result of noncompliance (ie, not wearing the Garmin watch), and the smaller data gaps may be due to movement artifacts or device malfunction. Scattered missing data due to movement artifacts is typically considered data missing at random or missing completely at random [16]. Data missing at random or missing completely at random do not necessarily produce a bias in outcomes [17]. Bursts of missing data that occur at similar times each day or routinely for a particular purpose (ie, bathing) may be considered data missing not at random, which could lead to biases in HRV outcomes [16]. Further understanding of the type of missing data within a 5-minute window is needed in future work.

Our secondary objective was to report validity and agreement between the Garmin photoplethysmography wrist-worn sensor compared with a chest-worn ECG sensor (Polar), which is closer to a gold standard that would be used in a laboratory setting to evaluate HRV [36,37] and has been used in the field for HRV biofeedback [38]. ICCs for agreement and consistency were mostly poor between Garmin and Polar devices. ICCs for consistency were moderate for median IBI and LF, suggesting these HRV metrics may be more reliable to measure in a real-world setting. Bland-Altman plots showed systematic bias in RMSDRR, HF, and LF/HF metrics. Specifically, Garmin tended to overreport lower values and underreport higher values, indicating that Garmin generally produced a smaller range of RMSDRR, HF, and LF/HF values compared to Polar. Our study compared processed HRV metrics (beyond a simple averaged heart rate calculation) between devices under free-living conditions. Previous studies have shown higher validity and agreement between wrist-worn photoplethysmography sensors and Polar ECG average heart rate [36,37]; however, these studies performed protocols in controlled, laboratory settings. Results from this study highlight that agreement and consistency between photoplethysmography wrist-worn sensors and ECG sensors are lower in free-living conditions and should be considered when evaluating HRV metrics. Commercially available wearable technology continues to grow at a rapid pace, and sensors that provide more accurate data are needed. Future studies should evaluate the accuracy of HRV data in emerging sensors.

Our study is the first to our knowledge to address the effects of missing data using real examples of HRV data patterns collected with photoplethysmography sensors; however, there are some limitations to our current work. In our current sample, we have limited labeling of HRV data. Future studies should include daily self-reported activity logs to determine when participants are sleeping, exercising, or not wearing their watch. We were unable to evaluate data missingness during levels of high activity (steps/min>100) because we did not have enough 5-minute windows with high step counts. It is best to evaluate high levels of activity in smaller time windows since many people engage in high activity for very short durations; however, a minimum window of 5 minutes is needed to accurately calculate frequency-domain HRV metrics [39]. In future work, we will aim to recruit individuals who are engaging in high levels of activity. In addition, we recruited healthy individuals for this study, but we did not exclude individuals with potential confounders that could have affected HRV data. For example, some participants may have had infrequent irregular heart rhythms (ie, heart skipping a beat) or may have taken medications that could have affected HRV values. Finally, our small sample size when comparing Garmin to Polar HRV data limits the generalizations we can make regarding Garmin's validity in real-world settings.

### Conclusion

Findings from this study have important implications for best practices for photoplethysmography wearables use in clinical and research settings. Time-domain HRV metrics (median IBI, STDRR, and RMSDRR) collected in a resting state remain stable until at least 35% data degradation. Frequency-domain HRV metrics (LF, HF, and LF/HF) are less resilient to missing data both at rest and during light activity and are unstable even at 10% data degradation. Studies that allow for greater than 10% data degradation for frequency-domain HRV metrics may introduce bias into their estimates, specifically the underestimation of LF and HF values. Correction methods such as gap-filling are possible to replace missing data; however, differences in HRV outcomes will still exist compared with more complete datasets without data loss [34]. In general, most derived HRV metrics from the photoplethysmography-based sensors used demonstrated moderate to poor agreement and consistency with ECG-based sensors in a real-world setting, and only median IBI values had reasonable agreement and consistency between the 2 sensing modalities. Despite the differences between photoplethysmography and ECG, we may conclude that given the stability of time-domain HRV metrics for up to 35% data degradation, these metrics could result in more reliable calculations of health metrics compared with frequency-domain metrics when monitoring patients using wrist-worn photoplethysmography sensors. In conclusion, while photoplethysmography sensors are valuable for remote monitoring of patients, future work is needed to identify best practices and the most accurate HRV metrics when using photoplethysmography sensors to evaluate HRV in medical settings.

## Abbreviations

COTS: commercial off the shelf
ECG: electrocardiogram
HF: high-frequency
HRV: heart rate variability
IBI: interbeat interval
ICC: intraclass correlation coefficient
LF: low-frequency
REDCap: Research Electronic Data Capture
RMSDRR: root-mean-square of the difference in successive IBI values
RTI: Research Triangle Institute
STDRR: standard deviation of interbeat interval values
Q-Q: Quantile-Quantile

## Data Availability

The data generated during and/or analyzed during this study are not publicly available due to restrictions of the Defense Advanced Research Projects Agency (contract 140D63-19-C-0023) but are available from the corresponding author on reasonable request.
